# Bradykinin β_2_ Receptor −58T/C Gene Polymorphism and Essential Hypertension: A Meta-Analysis

**DOI:** 10.1371/journal.pone.0043068

**Published:** 2012-08-10

**Authors:** Yan-yan Li, Hui Zhang, Jian Xu, Yun Qian, Xin-zheng Lu, Bing Yang, Minglong Chen, Zhi-jian Yang, Ke-jiang Cao

**Affiliations:** 1 Department of Geriatrics, First Affiliated Hospital of Nanjing Medical University, Nanjing, China; 2 Department of Cardiology, First Affiliated Hospital of Nanjing Medical University, Nanjing, China; Centers for Disease Control and Prevention, United States of America

## Abstract

**Background:**

Research has shown that bradykinin β_2_ receptor (*BDKRB2*) −58T/C gene polymorphism is correlated with the risk of essential hypertension (EH), but the results remain inconclusive.

**Objective and Methods:**

The objective of this study was to explore the association between *BDKRB2*−58T/C gene polymorphism and EH. A meta-analysis of 11 studies with 3882 subjects was conducted. Pooled odds ratios (ORs) for the association between *BDKRB2*−58T/C gene polymorphism and EH and their corresponding 95% confidence intervals (CIs) were estimated using the random effects model.

**Results:**

The *BDKRB2*−58T/C gene polymorphism was significantly correlated with EH under an allelic genetic model (OR = 1.24, 95% CI = 1.05–1.46; P = 0.01), a dominant genetic model (OR = 0.65, 95% CI = 0.47–0.90; P = 0.01), a recessive genetic model (OR = 1.146, 95% CI = 1.035–1.269; P = 0.009), a homozygote genetic model (OR = 1.134, 95% CI = 1.048–1.228; P = 0.002), and a heterozygote genetic model (OR = 1.060, 95% CI = 1.009–1.112; P = 0.019).

**Conclusions:**

The *BDKRB2*−58T/C gene polymorphism is associated with increased EH risk. The results of this study suggest that carriers of the −58C allele are susceptible to EH.

## Introduction

The kallikrein–kinin system (KKS) is an important hormonal system that takes part in blood pressure (BP) and renal sodium regulation [1]. Bradykinin is one of the strongest vasodilator substances, and it has powerful diuretic effects. It exerts its vasodilatory effects mainly by expanding the vessels directly, confronting the vasoconstrictive effects of angiotensin II and noradrenaline, and promoting the synthesis of endogenous vasodilator substances, such as nitric oxide.

The function of bradykinin is mediated by two receptor subtypes, namely, β_1_ (BDKRB1) and β_2_ (BDKRB2) [2]. BDKRB1 expression is low in healthy individuals; however, under such pathological states as inflammation and tissue injury, it could be upregulated. The physical effects of bradykinin are mostly mediated by BDKRB2. The *BDKRB2* gene, located in 14q32.1–32.2, spans approximately 4 kb and consists of three exons. In 1996, Braun et al. detected −58T/C variations in the *BDKRB2* promoter region and found that these could lead to a reduction in *BDKRB2* transcription, which might be associated with the pathogenesis of essential hypertension (EH) [3].

The relationship between *BDKRB2*−58T/C gene polymorphism and EH has been widely studied, but the results remain inconclusive. In 2000, Gainer et al. found that *BDKRB2*−58C might represent a susceptibility marker for EH in African Americans [4]. In 2006, Dong et al. found an association between *BDKRB2*−58T/C gene polymorphism and EH and concluded that the −58CC genotype is associated with increased EH risk in a Chinese population [5]. However, in 2012, Bhupatiraju et al. did not identify any such association in an Indian population [6]. We thus performed a meta-analysis of 11 studies, including 1947 patients with EH and 1935 control subjects, to deduce a reasonable conclusion on the relationship between *BDKRB2*−58T/C gene polymorphism and EH (Supplement S5).

**Figure 1:**
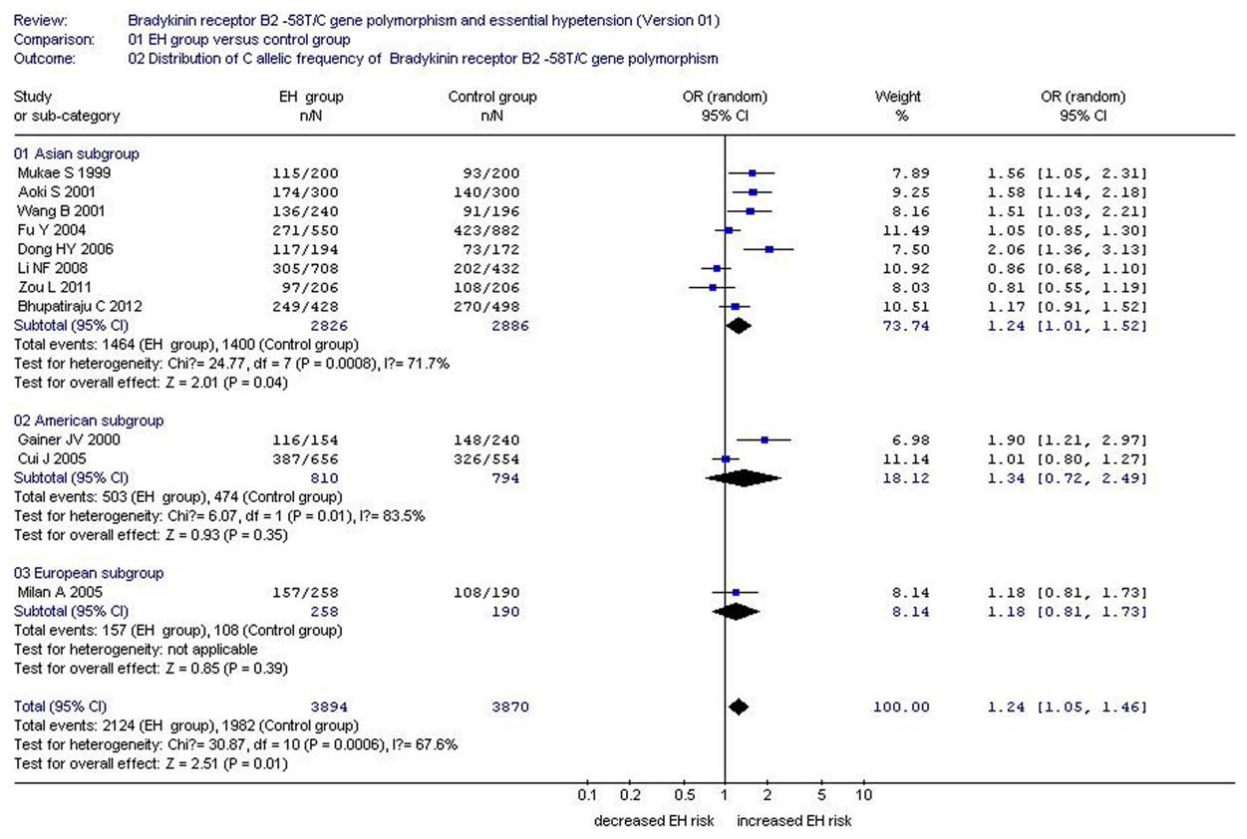
Forest plot of essential hypertension associated with *BDKRB2* -58T/C gene polymorphism under an allelic genetic model (distribution of C allelic frequency of *BDKRB2* gene).

## Materials and Methods

### Publication Search and Inclusion Criteria

PubMed, EMBASE, Web of Science, China Biological Medicine Database, and China National Knowledge Infrastructure were searched for relevant articles with the terms “essential hypertension”, “bradykinin β_2_ receptor”, and “polymorphism”. Studies published before 2012 were obtained (last research updated on July 7, 2012).

Studies that met the following major criteria were included: (a) the association between *BDKRB2*−58T/C gene polymorphism and EH was assessed; (b) the diagnosis of EH was in line with the 1999 diagnostic criteria of the World Health Organization in which systolic BP≥140 mmHg, diastolic BP≥90 mmHg, and treatment with antihypertensive medication defined EH, excluding patients with secondary hypertension, cardiomyopathy, valvular heart disease, congenital heart diseases, and renal failure; (c) the Hardy–Weinberg equilibrium (HWE) was followed; and (d) the results of the same data used in different studies were adopted only once. If articles with similar data were published by the same work group, the study with the larger sample size was selected.

**Figure 2:**
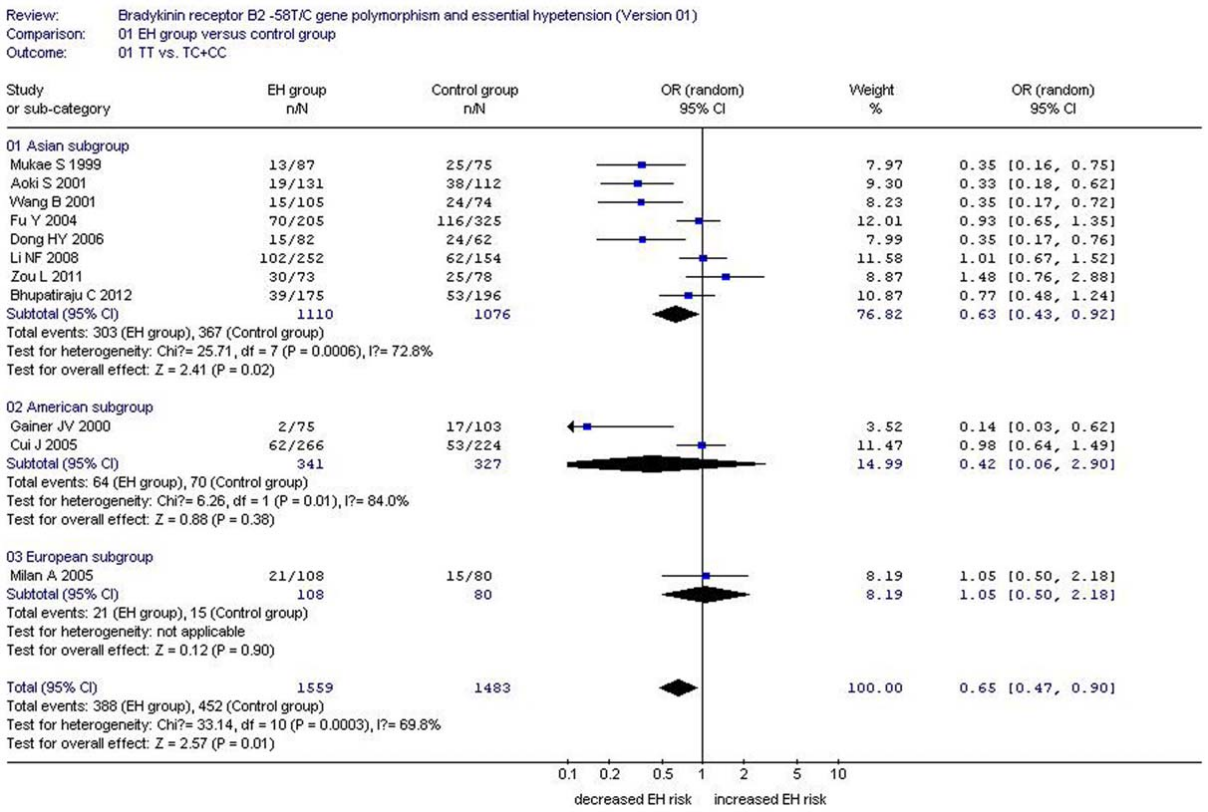
Forest plot of essential hypertension associated with *BDKRB2* -58T/C gene polymorphism under a dominant genetic model (TT/TC+CC).

### Data Extraction

The data were collected according to a standard protocol. Repeated publications, studies against the selection criteria, and work providing insufficient data were excluded from the meta-analysis. Data were recorded as follows: first author’s name, publication year, study region, number of genotypes, genotyping, study design, matching criteria, total number of case patients, and total number of control subjects.

### Statistical Analysis

Five genetic models (allelic, dominant, recessive, homozygote, and heterozygote) were used. The association between *BDKRB2*−58T/C gene polymorphism and EH reported under these models was analyzed using odds ratios (ORs) with 95% confidence intervals (CIs). Between-study heterogeneity was calculated by χ^2^-based *Q* analysis, and significance was set at P<0.05 [7]. The variation caused by heterogeneity was estimated by calculating the inconsistency index *I* ^2^. If heterogeneity among studies was detected, the DerSimonian–Laird random effects pooling method was used [8]; otherwise, the Mantel–Haenszel method fixed effects model was applied [9]. The *Z* test was used to determine the pooled OR, and significance was set at P<0.05.

**Figure 3:**
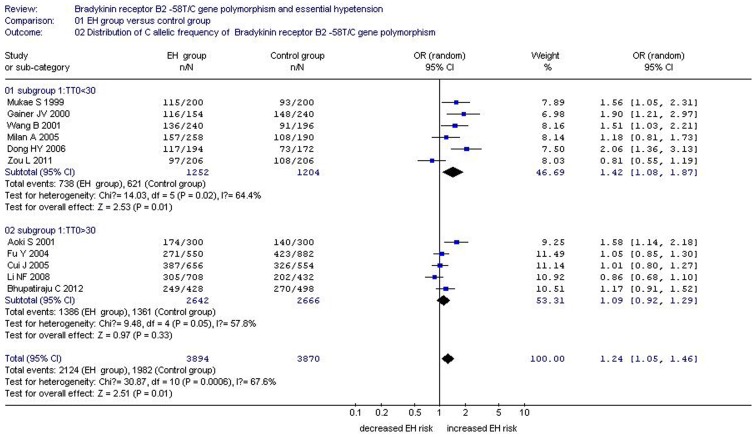
Forest plot of essential hypertension associated with *BDKRB2* -58T/C gene polymorphism under an allelic genetic model stratified by TT0 TT0: TT genotype sample size of control group.

Fisher’s exact test was used to assess the HWE, and P<0.05 was considered statistically significant. Potential publication bias was estimated by funnel plot analysis. The funnel plot asymmetry was assessed by Egger’s linear regression test on the natural logarithm scale of the OR (P<0.05) [10]. Statistical analysis was performed using STATA 11.0 (StataCorp, College Station, TX).

**Figure 4:**
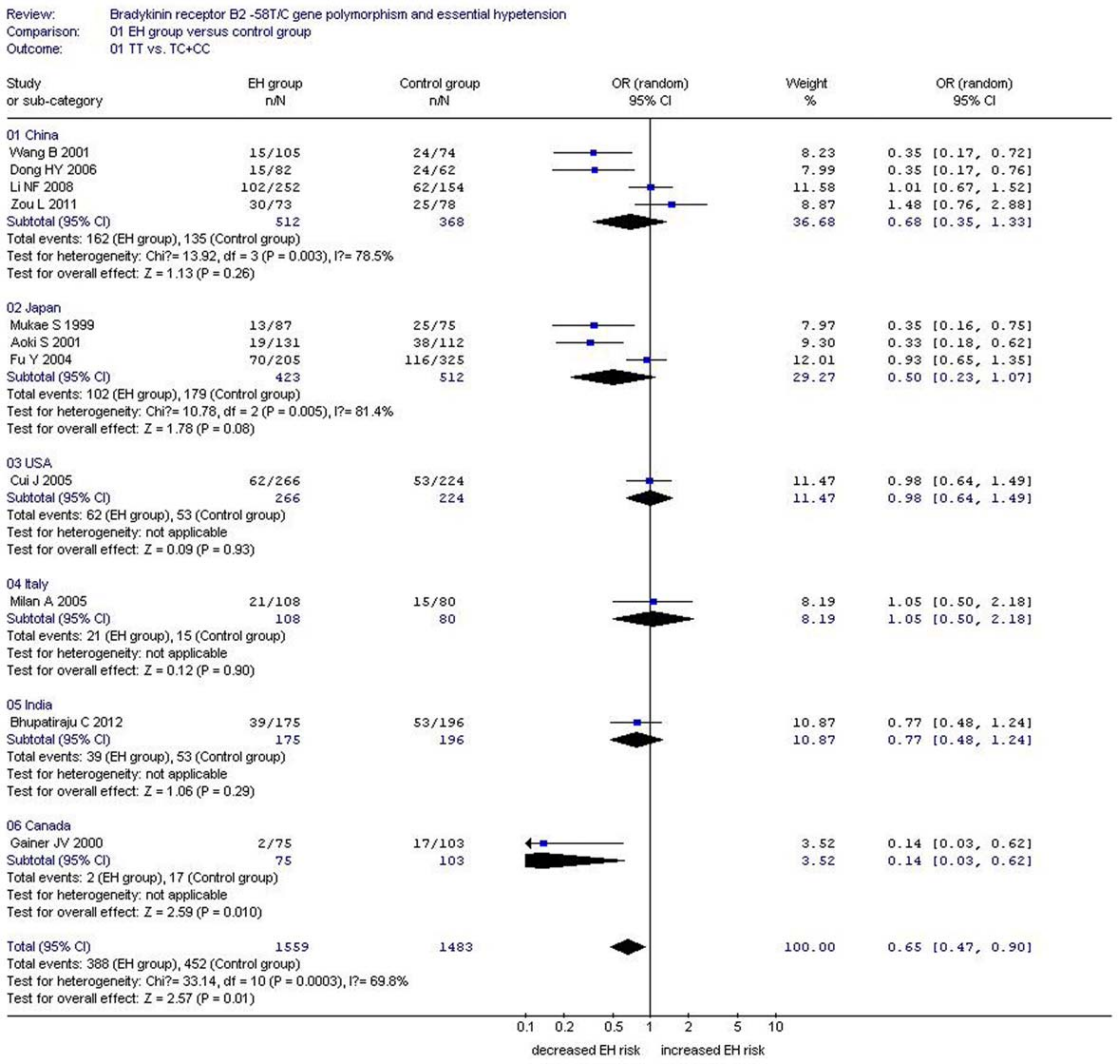
Forest plot of essential hypertension associated with *BDKRB2* -58T/C gene polymorphism under a dominant genetic model stratified by region.

## Results

### Studies and Populations

Our literature search yielded 20 relevant articles, 11 of which met the inclusion criteria. Of the 9 excluded studies, 2 were double publications, 2 were reviews, and 5 were not associated with the *BDKRB2*−58T/C gene polymorphism. No study was excluded for deviating from the HWE. Data were collected from 1947 patients with EH and 1935 control subjects (Supplement S1 and S6) [4]–[6], [11]–[18]. The six surveyed regions representing Asia, North America, and Europe included China, Japan, India, the United States, Canada, and Italy.

### Pooled Analysis

A significant association between *BDKRB2*−58T/C gene polymorphism and EH was found under the allelic (OR = 1.24, 95% CI = 1.05–1.46; P = 0.01), dominant (OR = 0.65, 95% CI = 0.47–0.90; P = 0.01), recessive (OR = 1.146, 95% CI = 1.035–1.269; P = 0.009), homozygote (OR = 1.134, 95% CI = 1.048–1.228; P = 0.002), and heterozygote (OR = 1.060, 95% CI = 1.009–1.112; P = 0.019) genetic models. Subgroup analysis stratified by continent also revealed a significant association between *BDKRB2*−58T/C gene polymorphism and EH in the Asian subgroup under the allelic (OR = 1.24, 95% CI = 1.01–1.52; P = 0.04), dominant (OR = 0.63, 95% CI = 0.43–0.92; P = 0.02), recessive (OR = 1.152, 95% CI = 1.010–1.315; P = 0.036), homozygote (OR = 1.166, 95% CI = 1.047–1.298; P = 0.005), and heterozygote (OR = 1.070, 95% CI = 1.011–1.132; P = 0.019) genetic models. No significant association was found in the American and European subgroups under any of the genetic models (P>0.05) (Supplement S2; Figures 1 and 2).

**Figure 5:**
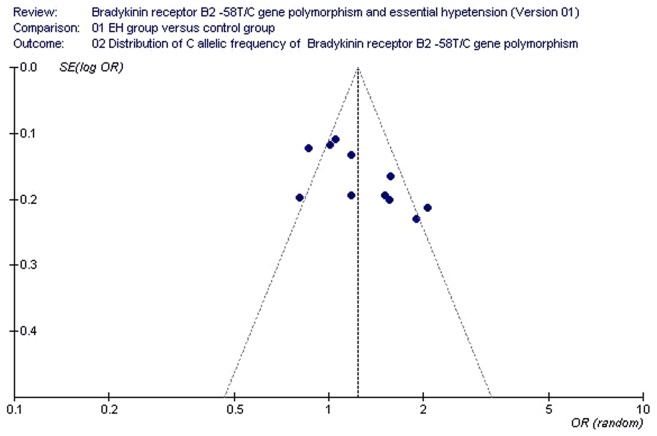
Funnel plot for studies of the association of essential hypertension associated with *BDKRB2* -58T/C gene polymorphism under an allelic genetic model (distribution of C allelic frequency of *BDKRB2* gene). The horizontal and vertical axis correspond to the OR and confidence limits. OR: odds ratio; SE: standard error.

In consideration of the significant heterogeneity we observed, a meta-regression was performed to explore the source of heterogeneity. Under the allelic genetic model, the heterogeneity could be explained by the number of control subjects with the TT genotype (TT0) (P = 0.036), total sample size of the control group (P = 0.037), and study region (P = 0.045). Based on TT0, the whole population was divided into two subgroups: Subgroup 1 was defined as TT0<30, whereas subgroup 2 was classified as TT0>30. In the subgroup analysis by TT0, under the allelic genetic model, significantly increased EH risk was detected in subgroup 1 (OR = 1.42, 95% CI = 1.08–1.87; P = 0.01, P_heterogeneity_ = 0.02). In subgroup 2, no significant increase in EH risk was found (OR = 1.09, 95% CI = 1.05–1.46; P = 0.33, P_heterogeneity_ = 0.05) (Supplement S2 and S3; Figure 3).

Under the dominant genetic model, the heterogeneity could be explained by the study region (P = 0.011), total sample size of the control group (P = 0.033), and TT0 (P = 0.041). The entire population was separated into six subgroups by study region (China, Japan, the United States, Italy, India, and Canada). A significant association was found in the Canadian subgroup (OR = 0.14, 95% CI = 0.03–0.62; P = 0.01), but not in the other five subgroups (P>0.05) (Supplement S2 and S4; Figure 4).

### Bias Diagnostics

Publication bias was assessed using funnel plot analysis and Egger’s test. The funnel plot did not show evidence of publication bias (Figure 5). Similarly, the absence of statistically significant differences in Egger’s test indicated that there was no publication bias in the current meta-analysis under the allelic genetic model (T = −0.78; P = 0.455).

## Discussion

This meta-analysis found a significant association between *BDKRB2*−58T/C gene polymorphism and EH: OR = 1.24 for the allelic genetic model, OR = 0.65 for the dominant genetic model, OR = 1.146 for the recessive genetic model, OR = 1.134 for the homozygote genetic model, and OR = 1.060 for the heterozygote genetic model. The subgroup analysis stratified by continent also revealed that the *BDKRB2*−58T/C gene polymorphism was significantly correlated with EH in the Asian subgroup (P<0.05), but not in the American and European subgroups (P>0.05). Such variation in the results can be attributed to the ethnic differences between groups.

In the following meta-regression to explore the heterogeneity source under an allelic genetic model, the confounding factors TT0, study region, and total sample size of the control group could partly explain the heterogeneity source, with TT0 being the most important factor. This finding suggested that the heterogeneity among the different studies can be ascribed to the nonuniformity in the sample size of control subjects with the TT genotype. Subgroup analysis according to the number of control subjects with the TT genotype revealed that EH risk was significantly increased only in the TT0<30 subgroup.

Research has shown that the *BDKRB2*−58T/C gene polymorphism is associated with increased EH risk, especially in Asians. Carriers of the −58C allele of the *BDKRB2* gene might be predisposed to developing EH. In 2010, Niu et al. performed a meta-analysis aiming to provide a comprehensive evaluation of the correlation between *BDKRB2*−58T/C gene polymorphism and EH. They found that the −58T allele exhibited a protective effect on hypertension only in African Americans (P = 0.04) and had no effect on hypertension in Asians and Caucasians (P>0.05) [19]. Their results differ from those we obtained in the present meta-analysis. Niu et al. included only 4 studies with 823 case patients and 916 control subjects, whereas we analyzed 11 studies with 1945 case patients and 1937 control subjects. Therefore, our results should be more reasonable than theirs.

EH is a polygenic disease caused by both environmental and hereditary factors [20]. Research has shown that the KKS plays an important role in regulating BP. As a vasoactive substance, bradykinin could adjust the release of a series of biological active media, such as prostaglandin, nitric oxide, and platelet-activating factor, thus causing vessels to dilate and the BP to drop and suppress the proliferation of smooth muscle cells, which exert a protective effect on the cardiovascular system [14]. BDKRB2 is a transmembrane G protein-coupled protein. It generally mediates most functions of the KKS. An abnormal gene structure of *BDKRB2* would contribute to EH and other cardiovascular diseases, suggesting that it is a candidate gene for EH. Bradykinin could lower renal vascular resistance and increase the renal blood flow volume, which in turn could increase urine sodium excretion [21]–[22]. *BDKRB2*−58T/C mutation could lower the transcription rate of the *BDKRB2* gene, constrict vessels, and create an imbalance between water and salt, which all contribute to EH. The results of the current meta-analysis have confirmed these findings.

This work was not without limitations. The large-scale studies on the relationship between EH and *BDKRB2*−58T/C gene polymorphism included in the meta-analysis still proved inadequate. BDKRB2 was affected not only by the *BDKRB2*−58T/C gene polymorphism but also by such environmental factors as tissue injury and inflammation. Lastly, the type of association between *BDKRB2*−58T/C gene polymorphism and EH could not be easily adjusted. All these represent a weakness of the study.

The results of this meta-analysis suggest that the *BDKRB2*−58C allele might increase EH risk. This conclusion provides a strong foundation for formulating a strategy for individual therapy in patients with EH. Given the abovementioned limitations, however, this finding warrants further investigation.

## Supporting Information

Supplement S1 **Characteristics of the investigated studies of the association between bradykinin β2 receptor -58T/C gene polymorphism and essential hypertension.**
(DOC)

Supplement S2 **Summary of meta-analysis of association of bradykinin β2 receptor -58T/C gene polymorphism and essential hypertension.**
(DOC)

Supplement S3 **The meta-regression results among 11 studies under an allelic genetic model for -58T/C gene polymorphism of bradykinin β2 receptor.**
(DOC)

Supplement S4 **The meta-regression results among 11 studies under a dominant genetic model for -58T/C gene polymorphism of bradykinin β2 receptor.**
(DOC)

Supplement S5 **PRISMA 2009 Checklist.**
(DOC)

Supplement S6 **PRISMA 2009 Flow Diagram.**
(DOC)
