# Supplementary material for: Bradykinin β2 Receptor −58T/C Gene Polymorphism and Essential Hypertension: A Meta-Analysis
Source: PLoS One. 2012 Aug 10;7(8):e43068. doi: 10.1371/journal.pone.0043068 (PMC3416764; doi:10.1371/journal.pone.0043068)
Supplement: Supplement S1 — Characteristics of the investigated studies of the association between bradykinin β2 receptor -58T/C gene polymorphism and essential hypertension. (DOC) [file pone.0043068.s001.doc]

**Supplement S1.Characteristics of the investigated studies of the association between *bradykinin β2 receptor* -58T/C gene polymorphism and essential hypertension**

| Author | Year | Region | Continent | EH | | | Control | |  | Geno-typing | Study design | Matching criteria | sample size (EH/control) |
| --- | --- | --- | --- | --- | --- | --- | --- | --- | --- | --- | --- | --- | --- |
|  |  |  |  | TT | TC | CC | TT | TC | CC |  |  |  |  |
| Mukae S [11] | 1999 | Japan | Asian | 13 | 59 | 28 | 25 | 57 | 18 | PCR | Case-control | Age,sex,ethnicity | 100/100 |
| Aoki S [12] | 2001 | Japan | Asian | 19 | 88 | 43 | 38 | 84 | 28 | PCR-SSCP | Case-control | NA | 150/150 |
| Wang B[13] | 2001 | China | Asian | 15 | 74 | 31 | 24 | 57 | 17 | PCR-SSCP | Case-control | Sex,ethnicity | 120/98 |
| Fu Y [14] | 2004 | Japan | Asian | 70 | 139 | 66 | 116 | 227 | 98 | PCR- RFLP | Case-control | Sex,ethnicity | 275/441 |
| Dong HY[5] | 2006 | China | Asian | 15 | 47 | 35 | 24 | 51 | 11 | PCR-SSCP | Case-control | Age,sex,BMI,ethnicity | 97/86 |
| Li NF[15] | 2008 | China | Asian | 102 | 199 | 53 | 62 | 106 | 48 | PCR-SSCP | Case-control | Age,sex, ethnicity | 354/216 |
| Zou L[16] | 2011 | China | Asian | 30 | 49 | 24 | 25 | 48 | 30 | PCR- RFLP | Case-control | Age,sex,BMI,ethnicity | 103/103 |
| Bhupatiraju C[6] | 2012 | India | Asian | 39 | 101 | 74 | 53 | 122 | 74 | PCR-SSCP | Case-control | Smokers,alcoholics | 214/249 |
| Gainer JV[4] | 2000 | Canada | American | 2 | 34 | 41 | 17 | 58 | 45 | PCR- RFLP | Case-control | Sex,ethnicity | 77/120 |
| Cui J[17] | 2005 | USA | American | 62 | 145 | 121 | 53 | 122 | 102 | PCR | Case-control | Sex,ethnicity | 328/277 |
| Milan A[18] | 2005 | Italy | European | 21 | 59 | 49 | 15 | 52 | 28 | PCR- RFLP | Case-control | Age,sex,BMI,ethnicity | 129/95 |

Abbreviations:

Year:publication year;

PCR-RFLP: polymerase chain reaction-restriction fragment length polymorphism

PCR-SSCP: single-strand conformation polymorphism

BMI: body mass index
