# Supplementary material for: Bradykinin β2 Receptor −58T/C Gene Polymorphism and Essential Hypertension: A Meta-Analysis
Source: PLoS One. 2012 Aug 10;7(8):e43068. doi: 10.1371/journal.pone.0043068 (PMC3416764; doi:10.1371/journal.pone.0043068)
Supplement: Supplement S2 — Summary of meta-analysis of association of bradykinin β2 receptor -58T/C gene polymorphism and essential hypertension. (DOC) [file pone.0043068.s002.doc]

**Supplement S2. Summary of meta-analysis of association of *bradykinin β2 receptor* -58T/C gene polymorphism and essential hypertension**

| **Genetic model** | **Ethnicity** | **Pooled OR (95% CI)** | **P value** | **Literature number** | **EH size** | **control size** | ***P*_heterogeneity_** |
| --- | --- | --- | --- | --- | --- | --- | --- |
| **allelic genetic model** | Asian subgroup | 1.24(1.01-1.52) | 0.04^﹡^ | 8 | 1413 | 1443 | 0.0008^﹡^ |
|  | American subgroup | 1.34(0.72-2.49) | 0.35 | 2 | 405 | 397 | 0.01^﹡^ |
|  | European subgroup | 1.18(0.95-1.22) | 0.39 | 1 | 129 | 95 | NA |
|  | subgroup1:TT0<30 | 1.42(1.08-1.87) | 0.01^﹡^ | 6 | 626 | 602 | 0.02^﹡^ |
|  | subgroup 2:TT0>30 | 1.09(0.92-1.29) | 0.33 | 5 | 1321 | 1333 | 0.05 |
|  | **whole population** | **1. 24(1.05-1.46)** | **0.01**^﹡^ | **11** | **1947** | **1935** | **0.0006**^﹡^ |
| **Dominant genetic model** | Asian subgroup | 0.63(0.43-0.92) | 0.02^﹡^ | 8 | 1413 | 1443 | 0.0006^﹡^ |
|  | American subgroup | 0.42(0.06-2.90) | 0.38 | 2 | 405 | 397 | 0.01^﹡^ |
|  | European subgroup | 1.05(0.50-2.18) | 0.90 | 1 | 129 | 95 | NA |
|  | China subgroup | 0.68(0.35-1.33) | 0.26 | 4 | 674 | 503 | 0.003^﹡^ |
|  | Japan subgroup | 0.50(0.23-1.07) | 0.08 | 3 | 525 | 691 | 0.005^﹡^ |
|  | USA subgroup | 0.98(0.64-1.49) | 0.93 | 1 | 328 | 277 | NA |
|  | Italy subgroup | 1.05(0.50-2.18) | 0.90 | 1 | 129 | 95 | NA |
|  | India subgroup | 0.77(0.48-1.24) | 0.29 | 1 | 214 | 249 | NA |
|  | Canada subgroup | 0.14(0.03-0.62) | 0.01 | 1 | 77 | 120 | NA |
|  | **whole population** | **0.65(0.47-0.90)** | **0.01**^﹡^ | **11** | **1947** | **1935** | **0.0003**^﹡^ |
| **recessive genetic model** | **whole population** | **1.146(1.035-1.269)** | **0.009**^﹡^ | **11** | **1947** | **1935** | **0.002**^﹡^ |
| **Homo genetic model** | **whole population** | **1.134(1.048-1.228)** | **0.002**^﹡^ | **11** | **1947** | **1935** | **0**^﹡^ |
| **Hetero genetic model** | **whole population** | **1.060(1.009-1.112)** | **0.019** | **11** | **1947** | **1935** | **0.221** |

**﹡P<0.05.**

**Abbreviations: CI:confidence interval; OR:odds ratio;EH size: the total number of EH cases; control size: the total number of control group.**

**TT0: TT sample size of control group; NA: not applicable; allelic genetic model:** distribution of C allelic frequency of *BDKRB2* gene; **Dominant genetic model:** TT/TC+CC; **recessive genetic model: CC/TC+TT; homo genetic model:** homozygote genetic model, **CC/TT; hetero genetic model:** heterozygote genetic model, **TC/TT.**
