# Supplementary material for: Bradykinin β2 Receptor −58T/C Gene Polymorphism and Essential Hypertension: A Meta-Analysis
Source: PLoS One. 2012 Aug 10;7(8):e43068. doi: 10.1371/journal.pone.0043068 (PMC3416764; doi:10.1371/journal.pone.0043068)
Supplement: Supplement S3 — The meta-regression results among 11 studies under an allelic genetic model for -58T/C gene polymorphism of bradykinin β2 receptor. (DOC) [file pone.0043068.s003.doc]

**Supplement S3. The meta-regression results among 11 studies under an allelic genetic model for -58T/C gene polymorphism of *bradykinin β2 receptor***

|  | Coefficient | Standard Error | T value | P value | 95% Confidence Interval |
| --- | --- | --- | --- | --- | --- |
| CC0 | -0.0338861 | 0.0192331 | -1.76 | 0.153 | -0.0872857～0.0195134 |
| TC1 | 0.0245545 | 0.0130071 | 1.89 | 0.132 | -0.011559～0.060668 |
| ET | -0.0128304 | 0.0076619 | -1.67 | 0.169 | -0.0341033～0.0084425 |
| TT0 | -0.1170286 | -0.1170286 | -3.1 | 0.036^﹡^ | -0.2217011～-0.0123561 |
| Region | -0.2170321 | 0.0755257 | -2.87 | 0.045^﹡^ | -0.4267251-0.0073391 |
| CTO | 0.0411861 | 0.013434 | 3.07 | 0.037^﹡^ | 0.0038873～0.078485 |
| cons | -0.0490954 | 0.2470454 | -0.2 | 0.852 | -0.7350034～0.6368125 |

﹡:P<0.05

Coefficient: regression coefficient. The regression coefficients are the estimated increase in the lnOR per unit increase in the covariates. CC0: CC genotype sample size of control group; TC1: TC genotype sample size of EH group; ET: total sample size of EH group; TT0: TT genotype sample size of control group; Region : study region; CTO: total sample size of control group; cons：constant item.
