# Supplementary material for: Bradykinin β2 Receptor −58T/C Gene Polymorphism and Essential Hypertension: A Meta-Analysis
Source: PLoS One. 2012 Aug 10;7(8):e43068. doi: 10.1371/journal.pone.0043068 (PMC3416764; doi:10.1371/journal.pone.0043068)
Supplement: Supplement S4 — The meta-regression results among 11 studies under a dominant genetic model for -58T/C gene polymorphism of bradykinin β2 receptor. (DOC) [file pone.0043068.s004.doc]

**Supplement S4. The meta-regression results among 11 studies under a dominant genetic model for -58T/C gene polymorphism of *bradykinin β2 receptor***

|  | Coefficient | Standard Error | T value | P value | 95% Confidence Interval |
| --- | --- | --- | --- | --- | --- |
| CC0 | 0.0543257 | 0.0227898 | 2.38 | 0.063 | -0.0042573～0.1129087 |
| Publication year | 0.0666741 | 0.0262971 | 2.54 | 0.052 | -0.0009246～0.1342729 |
| TT0 | 0.1368155 | 0.0500116 | 2.74 | 0.041^﹡^ | 0.0082567～0.2653744 |
| Region | 0.3922005 | 0.099603 | 3.94 | 0.011^﹡^ | 0.13616280.6482382 |
| CTO | -0.0538483 | 0.0184713 | -2.92 | 0.033^﹡^ | -0.1013302～-0.0063663 |
| cons | -133.8327 | 52.74038 | -2.54 | 0.052 | -269.4062～1.740733 |

﹡:P<0.05

Coefficient: regression coefficient. The regression coefficients are the estimated increase in the lnOR per unit increase in the covariates. CC0: CC genotype sample size of control group; TT0: TT genotype sample size of control group; Region : study region; CTO: control group total sample size; cons：constant item.
