# Supplementary material for: Bradykinin β2 Receptor −58T/C Gene Polymorphism and Essential Hypertension: A Meta-Analysis
Source: PLoS One. 2012 Aug 10;7(8):e43068. doi: 10.1371/journal.pone.0043068 (PMC3416764; doi:10.1371/journal.pone.0043068)
Supplement: Supplement S6 — PRISMA 2009 Flow Diagram. (DOC) [file pone.0043068.s006.doc]

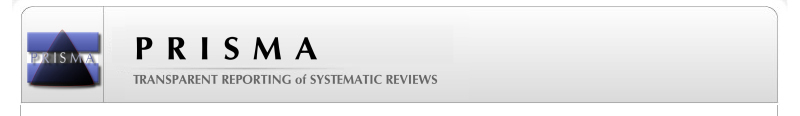
**PRISMA 2009 Flow Diagram**

**Screening**

**Included**

**Eligibility**

**Identification**

Records identified through database searching
(n =20 )

Additional records identified through other sources
(n =0 )

Records after duplicates removed
(n =18)

Records screened
(n =16 )

Records excluded for review characteristic
(n = 2 )

Full-text articles assessed for eligibility
(n =16)

Full-text articles excluded for deviation from HWE (n = 0 )

Studies included in qualitative synthesis
(n =11)

Records excluded for no association with BDKRB2 -58T/C gene polymorphism

(n =5 )

Records excluded for repeated publication
(n =2 )
